#  Knockout of the circadian gene, *Per2*, disrupts corticosterone secretion and results in depressive‐like behaviors and deficits in startle responses

**DOI:** 10.1186/s12868-020-00607-y

**Published:** 2021-01-28

**Authors:** Ashley L. Russell, Lauren Miller, Hannah Yi, Rita Keil, Robert J. Handa, T. John Wu

**Affiliations:** 1 Program in Neuroscience, Uniformed Services University of the Health Sciences, Bethesda, MD USA; 2 Center for Neuroscience and Regenerative Medicine, Uniformed Services University of the Health Sciences, Bethesda, MD USA; 3 Department of Obstetrics and Gynecology, Uniformed Services University of the Health Sciences, 4301 Jones Bridge Road, Bethesda, MD 20814 USA; 4 Department of Biomedical Sciences, Colorado State University, Fort Collins, CO USA

**Keywords:** Per2, Corticosterone, Circadian, Depression, Startle response

## Abstract

**Background:**

The Period Circadian Regulator 2 (*Per2*) gene is important for the modulation of circadian rhythms that influence biological processes. Circadian control of the hypothalamus-pituitary-adrenal (HPA) axis is critical for regulation of hormones involved in the stress response. Dysregulation of the HPA axis is associated with neuropsychiatric disorders. Therefore, it is important to understand how disruption of the circadian rhythm alters the HPA axis. One way to address this question is to delete a gene involved in regulating a central circadian gene such as *Per2* in an animal model and to determine how this deletion may affect the HPA axis and behaviors that are altered when the HPA axis is dysregulated. To study this, corticosterone (CORT) levels were measured through the transition from light (inactive phase) to dark (active phase). Additionally, CORT levels as well as pituitary and adrenal mRNA expression were measured following a mild restraint stress. Mice were tested for depressive-like behaviors (forced swim test (FST)), acoustic startle response (ASR), and pre-pulse inhibition (PPI).

**Results:**

The present results showed that *Per2* knockout impacted CORT levels, mRNA expression, depressive-like behaviors, ASR and PPI. Unlike wild-type (WT) mice, *Per2* knockout (*Per2*) mice showed no diurnal rise in CORT levels at the onset of the dark cycle. *Per2−/−* mice had enhanced CORT levels and adrenal melanocortin receptor 2 (*Mc2R*) mRNA expression following restraint. There were no changes in expression of any other pituitary or adrenal gene. In the FST, *Per2−/−* mice spent more time floating (less time struggling) than WT mice, suggesting increased depressive-like behaviors. *Per2−/−* mice had deficits in ASR and PPI startle responses compared to WT mice.

**Conclusions:**

In summary, these findings showed that disruption of the circadian system via *Per2* gene deletion dysregulated the HPA stress axis and is subsequently correlated with increased depressive-like behaviors and deficits in startle response.

## Background

The regulation of the hypothalamic-pituitary-adrenal (HPA) stress axis has evolved to be critically important for an organism’s survival. HPA axis dysregulation is associated with neuropsychiatric disorders, such as depression, anxiety, schizophrenia and post-traumatic stress disorder (PTSD) [1, 2]. An organism maintains homeostasis in response to a physical or psychological stressor through controlled regulation of the HPA axis. The paraventricular nucleus of the hypothalamus (PVN) integrates stress-related neuronal inputs to induce secretion of corticotropin-releasing factor (CRF) and arginine vasopressin (AVP) into the hypophyseal portal vasculature [3]. These neuropeptides bind to CRF receptor type 1 (CRFR1) stimulating the secretion of adrenocorticotropic hormone (ACTH) from anterior pituitary corticotrophs. ACTH, in turn, binds to the melanocortin receptor 2 (*Mc2R*) in the adrenal cortex inducing corticosterone (CORT) production [3, 4]. Acutely elevated CORT induces an inhibitory feedback mechanism to decrease the synthesis and release of CRF and ACTH, effectively shutting down further HPA axis activity [5]. Chronic, uncontrolled exposure to CORT and the subsequent dysregulation CORT release can have deleterious consequences on the brain and behavior leading to a number of disease states.

HPA axis activity is modulated by a central clock [6]. The main controller of the mammalian circadian clock is the suprachiasmatic nucleus of the hypothalamus (SCN). This clock is regulated by a set of genes (such as *Per2*) within the SCN work in negative transcription-translation feedback loops to stabilize and coordinate daily physiological rhythms including the diurnal CORT cycle [7]. Diurnal CORT fluctuations show peak levels prior to the active period (morning for humans, dark for rodents), declining levels throughout the course of the active period, and low levels during the inactive period [8]. This daily fluctuation is controlled by signals from the SCN to the paraventricular nucleus (PVN). The circadian system may also regulate the HPA axis by decreasing the sensitivity of the adrenal cortex to ACTH [6].

For this study, we focused on the role of a circadian gene, *Per2*, on the regulation of the HPA axis by utilizing a previously characterized *Per2* knockout (*Per2−/−*) mouse model [9, 10]. The *Per2* gene promoter region contains glucocorticoid response elements, which upregulate *Per2* expression, thereby directly linking the circadian pathways to the HPA axis [6, 11, 12]. Thus we wish to determine the impact of the *Per2* gene on the HPA axis. Furthermore, circadian disruption and HPA axis dysregulation have been shown to be related to depression and startle behaviors [20, 27, 28].

In the present study, we tested the hypothesis that deletion of the *Per2* gene, an essential regulator of the circadian clock [9, 10] disrupts the HPA axis and related behaviors, such as depression-like and startle/sensorimotor gating behaviors in mice. Therefore, we showed that *Per2−/−* mice lacked a diurnal rise in CORT and had a less sustained restraint-induced CORT response suggesting a disruption in the HPA axis. Additionally, *Per2−/−* mice had enhanced depressive-like behaviors and altered the startle response.

## Materials and methods

### Animals

Adult male *Per2−/−* mice (B6.Cg-*Per2*^*tm1Brd*^ *Tyr*^*c−Brd*^/J; Stock #003819) and their wild-type (WT) littermates (*Per2+/+)* were purchased from The Jackson Laboratory (Bar Harbor, ME). Mice were backcrossed to C57BL/6-Tyr^c−Brd^ mice to become homozygous for both the *Per2*^*tmBrd*^ target mutation and the recessive *Tyr*^*c−Brd*^ mutation (The Jackson Laboratory). *Per2+/+* littermates were used as WT controls. Animals were acclimated to the University vivarium for at least 10 days with daily handling prior to experiments. Animals were housed same sex 3–4 per cage. The mice were housed under controlled conditions of light (12 h light/12 h dark; lights off at ZT12; sudden light/dark transition) and temperature (24–25 ºC). Food and water were available *ad libitum*. All mice in the vivarium were housed in standard mouse shoebox cages and observed daily by the University veterinary staff as well as by investigative staff. Mice were tested between 2 and 5 months of age after the onset of puberty as determined by testicular descent. The distribution of animals into experimental groups was randomized. Separate cohorts of mice were used for each time point of time course study and for behavioral tests. All experiments were conducted in a separate procedure room (Experiments 1 and 2) or in the behavior core (Experiment 3). All animal procedures and care were conducted in accordance with the National Institute of Health Guide for Care and Use of Laboratory animals and approved by the Institutional Animal Use and Care Committee at the Uniformed Services University of the Health Sciences.

### Experimental design

**Fig. 1:**
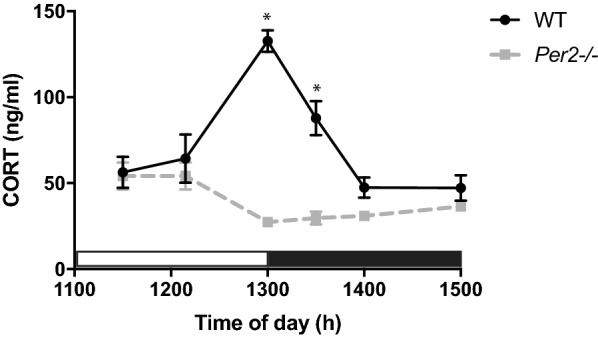
Effect of genotype (WT vs. *Per2−/−*) on the diurnal CORT rhythm. Peak CORT levels were observed in WT mice (solid black line) at the transition from lights-on to lights-out (Lights out at 1300 h or ZT12). There was no diurnal CORT rise in *Per2−/−* mice (dotted gray line). *p < 0.05 WT vs. respective *Per2−/−* time point (n = 6–15 animals per group)

Experiment 1 evaluated the effect of *Per2−/−* on diurnal CORT levels. WT control and *Per2−/−* mice were randomly assigned to one of six testing points in which CORT levels were measured: ZT1030 (WT n = 11, *Per2−/−* n = 14), ZT1115 (WT n = 6, *Per2−/−* n = 14), ZT12 [(onset of dark period), (WT n = 10, *Per2−/−* n = 15), ZT1230 (WT n = 10, *Per2−/−* n = 10), ZT13 (WT n = 9, *Per2−/−* n = 10) and ZT14 (WT n = 12, *Per2−/−* n = 13) (Fig. 1). These timepoints spanned the transition from the inactive (light) to active (dark) period. Each animal was tested at one timepoint. At the designated timepoint, one cage of animals (each in the same timepoint) were deeply anesthetized with CO_2_ over-inhalation and euthanized by decapitation. CO_2_ over-inhalation was induced by a flow rate that displaced 20% of the chamber volume per minute by introducing 100% CO_2_. Deep anesthesia is tested with toe pinch—a lack of response indicated deep anesthesia. Animals remained in the CO_2_ chamber for approximately 3–5 min. Trunk blood was collected into a 2 ml microcentrifuge tube within +/− 5 min of the stated time point. Blood was allowed to clot on ice for 15–30 min and serum was harvested by centrifugation (2000*g* for 15 min) and stored at − 80 ºC until use.

**Fig. 2:**
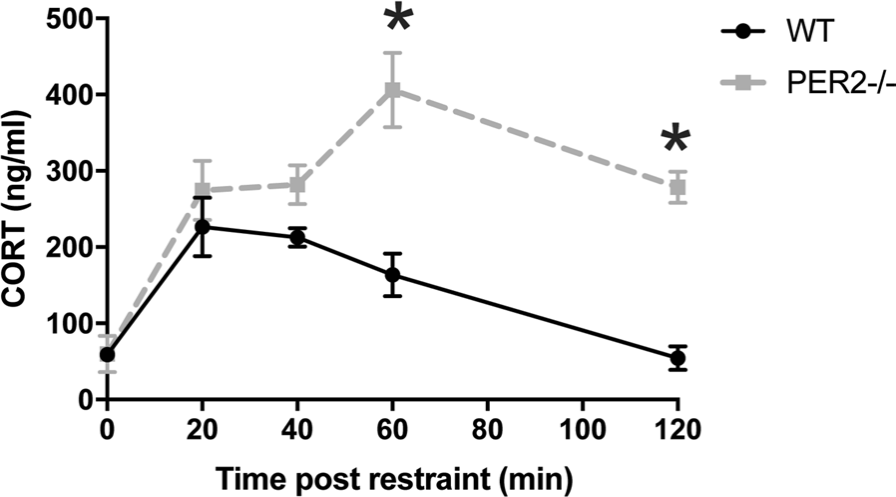
Effect of genotype (WT vs. *Per2−/−*) and restraint stress on CORT levels. Mice were restrained for 20 min (0–20 min; striped bar on x axis). In WT mice (solid black line), CORT peaked at 20 min, immediately at the conclusion of restraint and returned to baseline by 120 min. Similarly, CORT levels in the *Per2−/−* mice (dotted gray line) were elevated at 20 min (end of restraint stress), but unlike WT mice, CORT levels remained elevated throughout the 120 min time point. *p < 0.05 WT vs. respective *Per2−/−* time point. (n = 7–11 animals per group)

Experiment 2 evaluated the effect of restraint on CORT secretion in WT (n = 7–8 per timepoint) and *Per2−/−* mice (n = 7–11 per timepoint) during the light period (ZT7-ZT9; at 3–5 h before lights out). Mice were randomly assigned to one of five groups to account for each timepoint post restraint. Each cage of animals was assigned to one timepoint post restraint and each animal was only tested once due to testing procedures. Mice were not restrained (0 min time point) or restrained for 20 min using a Plexiglass restrainer (3.81 × 10.16 cm; Stoelting Co, Wood Dale, IL) and returned to their home cages until euthanized. Each animal was tested at only one of the following post-restraint timepoints: 0 min (no restraint), 20 min (immediately at the end of restraint), 40 min, 60 min or 120 min after the start of a 20 min restraint (Fig. 2). All mice were restrained in their home cages. Similar to Experiment 1, all animals were euthanized by CO_2_ over-inhalation and trunk blood was collected, stored on ice for 15–30 min, centrifuged to harvest serum, and stored at − 80 ºC until use (see above). Adrenals and pituitary were collected and snap frozen in 2-methyl butane (− 40 °C) on dry ice and stored at − 80 ºC until use.

Experiment 3 evaluated the effect of WT and *Per2−/−* on depressive-like behaviors, acoustic startle response (ASR) and pre-pulse inhibition (PPI) startle response. A separate cohort of animals was used for behavioral testing to avoid any influence of testing on CORT secretion. The completion of each behavioral test, animals were given at least one week until tested on the next assay. Prior to testing, animals were acclimated in their home cages to the testing room for 1 h. Behavioral assays were conducted during the inactive period (light period). All behaviors were video-recorded and scored blind to condition.

### CORT quantification

Serum CORT levels were analyzed using an enzyme-linked immunosorbent assay (ELISA) following the manufacturer’s instructions (Corticosterone Kit, Cayman Chemicals, Inc, Ann Arbor, MI). The inter-assay and intra-assay CV were 10.5% and 3.7%, respectively.

### Tissue collection and gene expression

Ribonucleic acid (RNA) was extracted from pituitaries (WT n = 6; *Per2−/−* n = 6) and adrenals (WT n = 8; *Per2−/−* n = 7) using previously established protocols [13, 14], Whole pituitaries and adrenals were placed in 1 mL Ribozol (Thermo Fisher Scientific, Waltham, MA) and homogenized and total RNA was isolated. Pelleted RNA was resuspended in nuclease-free water and treated with deoxyribonuclease I (Promega Corporation, Madison, WI). Standard sodium acetate (Sigma-Aldrich Inc, St. Louis, MO) precipitation and successive ethanol washes were performed for RNA purification. Pelleted RNA was dried and re-suspended in nuclease-free water. The concentration and purity of RNA were determined by measuring absorbance at 260 nm and 280 nm and the 260/280 ratio was calculated using a spectrophotometer (NanoDrop Lite spectrometer, Thermo Fisher Scientific). Within each extraction, random samples were run on 1% agarose gels to confirm sample integrity before reverse transcription. Total RNA was reverse transcribed using the Maxima First Strand cDNA Synthesis Kit (Thermo Fisher Scientific). In parallel, samples were also processed with no reverse transcription to assess for genomic contamination during PCR reactions.

Following previously established protocols, expression of mRNAs was measured using qPCR [13, 15], Primer sequences for measured genes are listed in Table 1. Two ng of cDNA template was amplified using iQ SYBR Green Supermix (BioRad Laboratories, Hercules, CA). Each total RNA sample was sequentially denatured for 10 sec at 95 °C, annealed for 30 sec at 60 °C, and extended for 30 sec at 72 °C for 40 cycles using the CFX Connect Real-Time System (BioRad). Reactions were performed in triplicate and normalized to either Beta-Actin (adrenal genes) or GAPDH (pituitary genes). The Delta Delta Ct (2^−ΔΔCT^) method was utilized to determine fold changes between WT and *Per2−/−* samples [16]. Melt curve analysis was performed at the end of each run to confirm single amplicon production.

**Table 1:** Relative gene expression in the adrenal (*Mc2R* and *Hsd1*) and pituitary (*Crfr1*) in WT and *Per2−/−* mice measured at lights on (1300 h)

Gene	Accession #	Primer sequence	WT	*Per2−/−*
Adrenal				
MCR2	NM_0010839	(F) 5’-ACACCAATGACACCGCAAGA-3’ (R) 5’-ACAGACTGCCCAACATGTCA-3’	*1.0 ± 0.2*	*1.3 ± 0.4*
HSD1	NM_008288.2	(F) 5’-AGCCGCACTTATCTGAAGCC-3’ (R) 5’-TTCCCTGGAGCATTTCTGGTC-3’	*1.0 ± 0.3*	*0.7 ± 0.3*
Pituitary				
CRFR1	NM_007762	(F) 5’-CAAAGTGCACTACCACATTGCCGT-3’ (R) 5’-TGAAAGCCGAGATGAGGTTCCAGT-3’	*1.0 ± 0.2*	*0.7 ± 0.3*

There were no differences in gene expression between WT and *Per2−/−* tissues (*p* > 0.05; n = 5–8 animals per genotype). Primer information is also provided

### Open field activity (OFA)

Locomotor activity was measured in WT (n = 16) and *Per2−/−* (n = 9) mice during the inactive period (light period, between ZT730 and ZT10) and conducted using a 40 cm × 40 cm open field apparatus (Stoelting Co). Light intensity was 80 lux in the arena center. Animals were allowed to roam freely for 60 min. Behavioral measures recorded include total distance traveled, time and entries in center/periphery and mean speed to determine overall activity. Each testing session was performed on up to eight mice from the same experimental group, each in a separate OF arena.

### Acoustic startle response and pre‐pulse inhibition (ASR and PPI, respectively)

Previous studies have shown correlations between startle reflex and sensorimotor gating and diurnal CORT [27, 28]. However, it is unknown how a dysregulated HPA axis may affect this behavior. To measure startle reflex and sensorimotor gating, ASR and PPI were used based on previous established protocols [17, 18]. WT (n = 16) and *Per2−/−* (n = 16) mice were individually acclimated to the test chamber for 5 minutes before testing began. Startle responses were measured using a weight sensitive platform inside of an acoustic startle response test system with sound attenuated chambers and speakers (Med Associate, Georgia, VT). Startle responses to a 110 or 120 dB tone were measured for ASR. In the PPI assay, 110 or 120 dB tones were preceded by either a 64 or 82 dB pre-pulse tone. Startle response and pre-pulse inhibition were tested concurrently. The 6 different tone combinations (64/110 dB, 64/120 dB, 82/110 dB, 82/120 dB, 110 dB, and 120 dB) were presented in a random order with an average 15 s between tones (range of 5–30 s) for a total of 32 trials. The pre-pulse tones were 20 secs long and the pulse/startle tones were 40 secs long. Four mice from the same cage were individually tested in each testing session of ASR/PPI paradigms.

### Porsolt’s forced swim test (FST)

Depression-like behavior was tested using the FST. Here, WT and *Per2−/−* (n = 12/genotype) mice were tested between ZT7 and ZT9 (light period). All mice were tested in individual chambers. A plastic cylindrical container (40 cm height × 27 cm diameter) was filled with 30 cm of 25 ºC water. Water was changed and cylinders were thoroughly washed with 70% ethanol between animals. Light intensity in the chamber was 80 lux. Animals were placed in the water for six min, allowed to acclimate to the assay prior to testing, and behaviors were analyzed during the last five min of testing. After completion of the test animals were dried with a clean towel and placed on a drying pad until dry. Behaviors analyzed included: time spent struggling and time spent immobile. Struggling was defined as rapid movement of the forelimbs that breaks the surface of the water and/or attempting to climb against the wall of the container. Immobility was defined as absence of any movement except for slight movements necessary for the animal to keep its head above water [19].

### Statistical analysis

CORT and mRNA expression data were analyzed by two-way analysis of variance (ANOVA) with genotype and time as main factors followed by Sidak’s *posthoc* comparisons where appropriate using SPSS and GraphPad statistical softwares. Behavioral measures (OFA, FST, ASR and PPI) were analyzed by unpaired Student’s t-test. Significance was accepted at p < 0.05. All values are reported as mean +/− SEM. Significant outliers were determined by the Grubb’s test with an alpha = 0.05. Any animal that was identified as an outlier in any experiment was removed from the study.

## Results

### *Per2−/−* mice lack a diurnal rise in CORT at the onset of the active period

The influence of *Per2* on the diurnal rise in CORT was tested during the transition from inactive (light period) to active (dark period) periods in WT and *Per2−/−* mice (Fig. 1). A two-way ANOVA revealed a main effect of time of day [F(2.892, 55.52) = 11.81, p < 0.05, n = 6–15 animals per group], a main effect of genotype [F(1, 25) = 41.21, p < 0.05] and a significant interaction between time of day × genotype [F(5, 96) = 21.06, p < 0.05]. As expected, WT CORT levels peaked at the onset of the active period (ZT12) and decreased to basal levels by ZT13. Basal levels are defined by the ZT1030 time point. In contrast, *Per2−/−* mice did not have diurnal fluctuation in CORT levels in that CORT levels remained the same throughout all time points.

### *PER2−/−* mice have enhanced CORT levels after restraint

We next tested if the HPA axis reactivity is also altered. WT and *Per2−/−* mice were exposed to a 20 min restraint. CORT was measured in samples collected at the onset of restraint and every 20 min up to 120 min after the end of restraint (Fig. 2). A two-way ANOVA revealed a main effect of time post-restraint [F(2.656, 34.53) = 20.09, p < 0.05, n = 7–11 animals per group], a main effect of genotype [F(1,17) = 21.94, p < 0.05], and a significant interaction between time post-restraint x genotype [F(4, 52) = 7.779, p < 0.05]. As expected, CORT was significantly elevated at 20 and 40 min following the onset of restraint and returned to baseline by 120 min. Peak CORT levels were observed at 20 min post restraint onset. *Per2−/−* mice also had elevated CORT in response to restraint, but the overall temporal profile differed compared to WT. In *Per2−/−* mice, CORT peaked at 60 min following onset and remained high throughout the recovery period (up to 120 min post restraint onset). CORT levels did not return to baseline (0 min) levels during the testing period.

### *Per2−/−* mice gene expression in the adrenal and pituitary

**Fig. 3:**
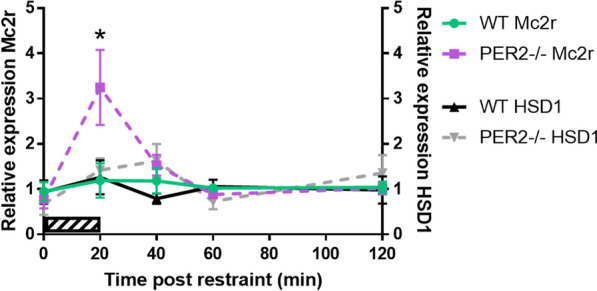
Effect of genotype on *Mc2R* and *Hsd1* gene expression in the adrenal. Mice were restrained for 20 min (0–20 min; striped bar). There was no effect of genotype on *Hsd1* expression in the adrenal. However, *Mc2R* expression increased in the adrenal immediately following restraint in *Per2−/−* mice, but not in WT mice. *p < 0.05 WT vs. respective *Per2−/−* time point. (n = 5–8 animals per group)

To determine if the observed disruptions in CORT levels in *Per2−/− mice* were due to alterations in the HPA axis, genes relevant to the HPA axis located in the adrenal (*Mc2R and HSD1*) and pituitary (*CRFR1)* were analyzed. There was no effect of genotype on pituitary *Crfr1*, adrenal *Hsd1* or *Mc2R* gene expression (Table 1). Expression of these genes relevant to HPA axis reactivity in WT and *Per2−/−* mice was also measured in response to a 20 min restraint. There were also no effects of genotype or restraint on adrenal *Hsd1* (Fig. 3). Interestingly, there was a main effect of time post-restraint onset [*F*(1.566, 14.88) = 5.530, *p* < 0.05, n = 5–8 animals per group] and a significant interaction between time post-restraint and genotype [F(4, 38) = 3.971, *p* < 0.05] for adrenal *Mc2R* gene expression. There was a trending, but not significant, [F(1, 13) = 3.689, *p* = 0.0770] main effect of genotype on *Mc2R* expression (Fig. 3). In WT mice, *Mc2R* mRNA levels did not change as a result of restraint. However, in *Per2−/−* mice, *Mc2R* mRNA expression peaked at 20 min after the onset of restraint and returned to baseline for subsequent time points. Additionally, there was no difference in CRFR1 levels after restraint between WT and *Per2−/−* mice (Table 2).

**Table 2:** Effect of genotype (WT v *Per2−/−*) on the CRFR1 mRNA expression in the pituitary following a 20 min restraint

	WT	*Per2−/−*
0 min	1.1 ± 0.2	0.6 ± 0.2
20 min	1.2 ± 0.3	1.2 ± 0.2
40 min	1.2 ± 0.4	1.0 ± 0.4
60 min	1.1 ± 0.2	1.2 ± 0.3
120 min	1.0 ± 0.2	1.1 ± 0.3

Measurements were taken before restraint (0 min), immediately following restraint (20 min) and 40, 60, and 120 min from the onset of restraint. There were no differences between *Per2−/−* and WT mice in CRFR1 mRNA expression levels (n = 5–7)

### *Per2−/−* mice have normal gross locomotor activity

**Fig. 4:**
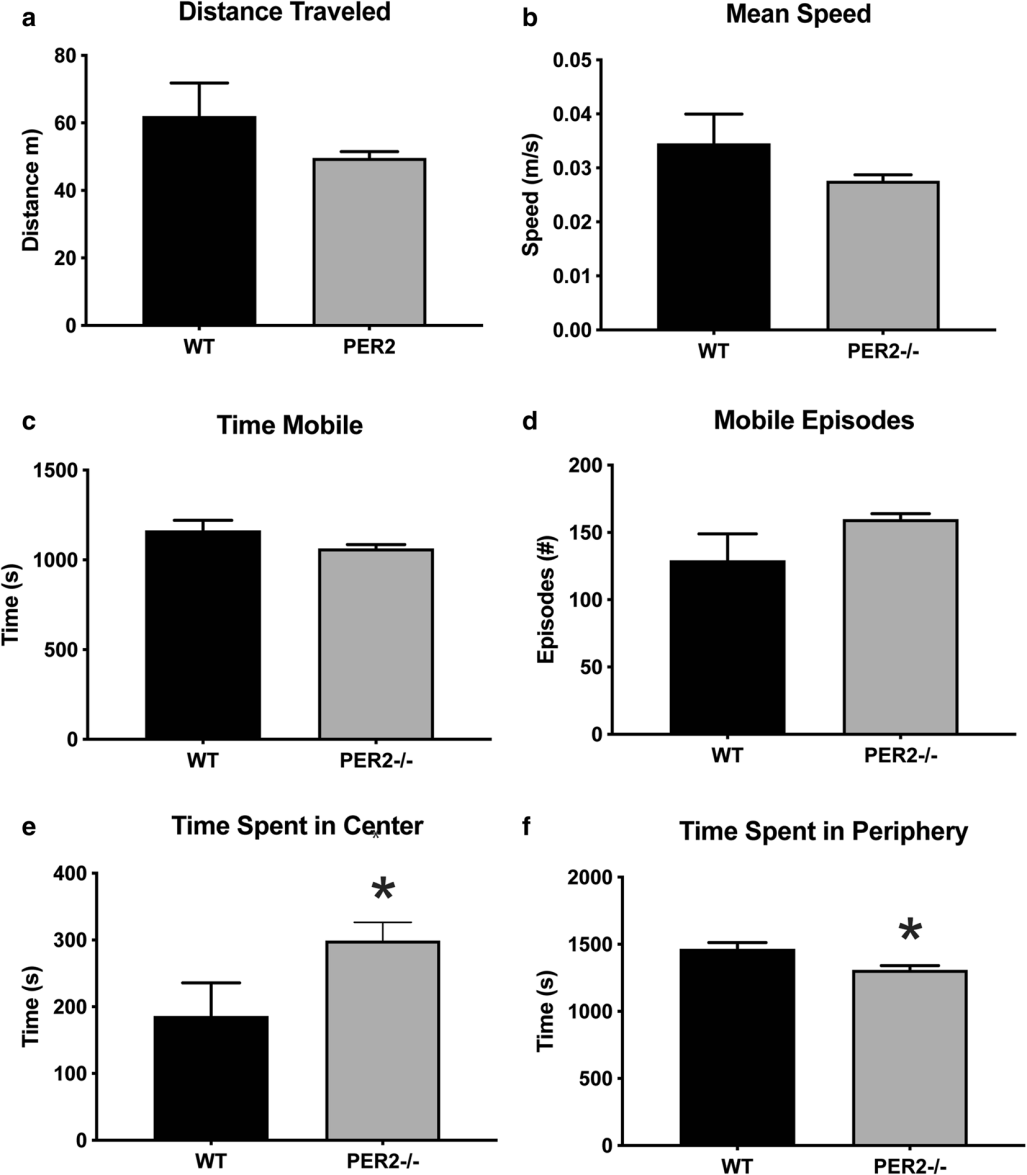
Effect of genotype (WT vs. *Per2−/−*) on open field activity. There was no effect of genotype on any measures taken during the open field activity. WT and *Per2−/−*** a** traveled the same distance,** b** traveled at the same speed,** c** spent the same time mobile (moving around the arena), and** d** had the same number of mobile episodes. *Per2−/−* mice spent** e** more time in the center and** f** less time in the periphery compared to WT mice. *p < 0.05 WT vs. *Per2−/−* mice. (n = 9–16 animals per group)

To study the effect of *Per2−/−* on locomotor activities, behaviors in an open field arena were measured (Fig. 4a–d). Unpaired t tests revealed no effect of genotype on distance traveled [t(22) = 1.681, *p* > 0.05, n = 9–16 animals per group], mean speed [t(22) = 1.675, *p* > 0.05], mobile time [t(22) = 1.913, *p* > 0.05], or mobile episodes [t(22) = 2.054, *p* > 0.05]. This suggests that any difference in subsequent behavioral measures is not due to changes in overall activity levels nor ability to move.

### *Per2−/−* mice have decreased anxiety-like behaviors

To examine the effect of *Per2−/−* on anxiety-like behaviors we tested mice in the OFA during the light period and measured time spent in the center and periphery of the arena (Fig. 4e, f). Unpaired t tests revealed a significant effect of genotype on time spent in the center [t(22) = 2.153, *p* > 0.05, n = 9–16 animals per genotype] and periphery [t(22) = 2.687, *p* < 0.05]. Overall, *Per2−/−* mice spent more time in the center of the arena and less time in the periphery compared to WT mice suggesting less anxiety-like behaviors.

### *Per2−/−* mice have increased depressive-like behaviors

**Fig. 5:**
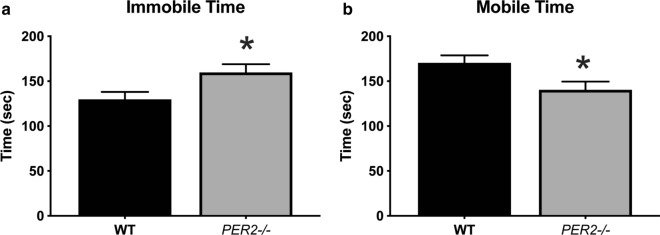
Effect of genotype on behaviors in the Forced Swim Test measured during the inactive period. *Per2−/−* mice **a** spent less time mobile (struggling/swimming) and **b** more time immobile (floating) compared to WT mice. *p < 0.05 WT vs. *Per2−/−* mice. (n = 12 per group)

Variations in the Per2 genes are connected to depression vulnerability in humans [20]. Therefore, we wanted to determine if knockout of *Per2* mimics this is in mice by testing for depressive-like behaviors using the FST during the light period (Fig. 5). An unpaired t test showed a significant change in mobile time [t(22) = 2.401, *p <* 0.05, n = 12 animals per group] and immobile time [t(22) = 2.401, *p* < 0.05]. *Per2−/−* mice spent less time mobile (swimming/struggling) and more time immobile (floating) compared to WT mice, indicative of increased depressive-like behaviors.

### *Per2−/−* mice have an altered startle response

**Fig. 6:**
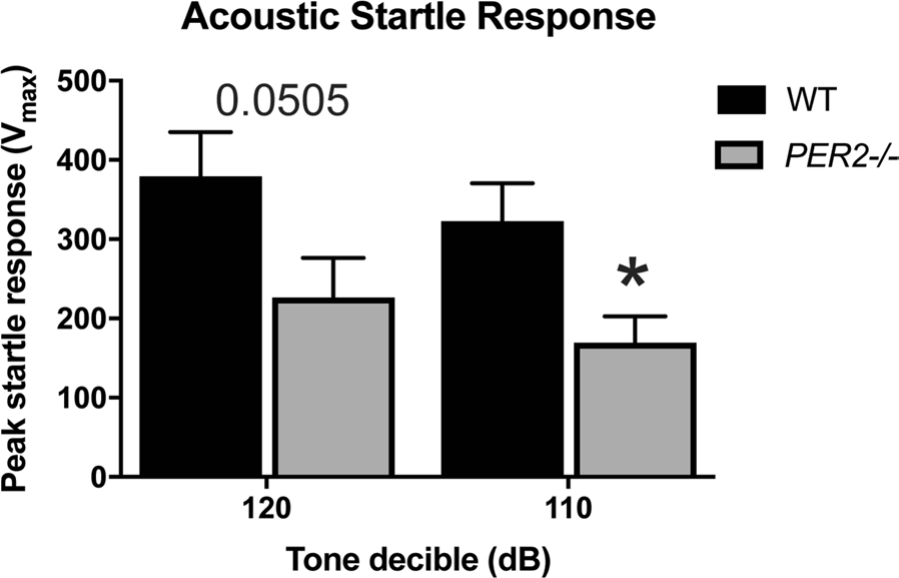
Effect of genotype on acoustic startle response. *Per2−/−* animals have decreased acoustic startle response when compared to the WT controls to both the 120 dB and the 110 dB tones. *p < 0.05. (n = 16 animals per genotype)

**Fig. 7:**
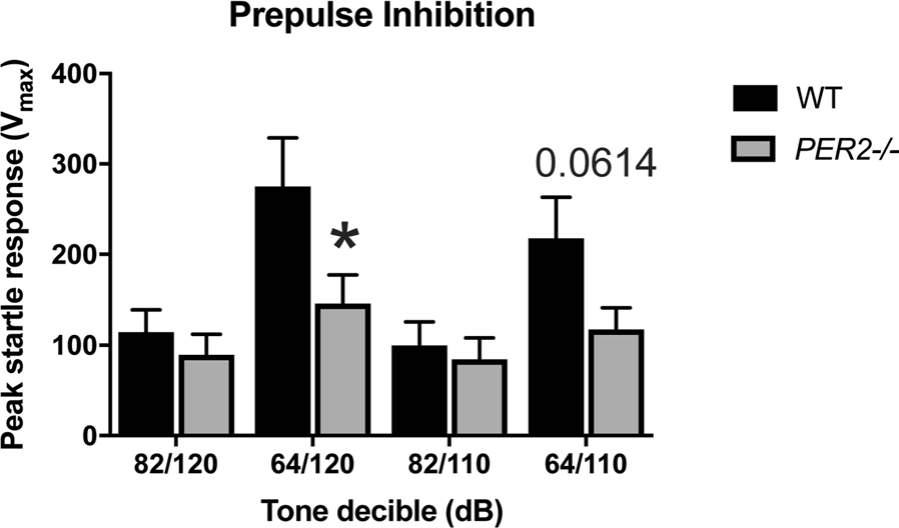
Effect of genotype on startle response after a pre-pulse tone. *Per2−/−* animals had decreased startle response compared to WT mice, but only when the initial pre-pulse is more distinct from the second tone (64/120 and 64/110 dB). There was no effect when the two tones were more similar (i.e. 82/120 and 82/110). *p < 0.05 WT compared to the *Per2−/−* counterpart. (n = 16 animals per group)

To measure startle and sensorimotor gating in *Per2−/−* mice, the ASR and PPI assays were used (Figs. 6 and 7, respectively). An unpaired t test revealed a significant effect of startle response to the 110 dB tone [t(30) = 2.63, *p* < 0.05, n = 16 mice per group] and a trending, but not significant, effect at the 120 dB tone [t(30) = 2.038, *p* = 0.0505]. *Per2−/−* mice startled less in response to a tone compared to WT controls, as measured by the peak startle response (Fig. 6). In the PPI assay, an unpaired t test revealed a significant effect at 64/120 dB pre-pulse pairing [t(30) = 2.072, *p* < 0.05, n = 16 mice per group], and a trending, but non-significant effect, at 64/110 pre-pulse pairing [t(30) = 1.943, p = 0.0614]. There was no effect at the 82/110 dB pre-pulse pairing [t(30) = 0.4313, *p* = 0.6694] and the 82/120 dB pre-pulse pairing [t(30) = 0.731, *p* = 0.4707]. *Per2−/−* mice had decreased PPI-induced peak startle response compared to WT controls in cases where the pre-pulse tone was more distinct (further dB) from the second tone (Fig. 7). Overall, these data show deficits in startle response in *Per2−/−* mice.

## Discussion

Dysregulation of the HPA axis by disrupting circadian rhythmicity is implicated in the pathophysiology of psychiatric disorders [21]. However, the interactions between the circadian clock and HPA axis circuitry are not well understood. We address the relationship between circadian clock regulation and the HPA axis using the mouse with a *Per2* gene deletion. In the present study, deletion of *Per2* alters the HPA axis and impairs related behavioral function. These experiments revealed that the Per2 gene is involved in the diurnal rise of CORT and in the recovery of CORT levels to baseline after a mild psychogenic stressor.

Communication between the circadian system and the HPA axis has been previously established. The SCN is connected to the HPA axis through its projections to CRF/AVP-containing neurons of the PVN as well as through its modulation of the sensitivity of the adrenal cortex to ACTH [6]. SCN lesions abolish the diurnal rhythm of ACTH and CORT [7, 22, 23]. Our findings complement previous work using *Per2−/−* mice which show an attenuated diurnal CORT rise during the transition from the inactive (light) to the active (dark) period [12]. However, our studies do not address the question of HPA axis rhythmicity. A thorough examination of CORT levels throughout the course of a day, including the transition from dark (active) to light (inactive) periods is needed to understand how the circadian clock may affect HPA axis rhythmicity. Additionally, these studies cannot identify the site of disruption since *Per2* was knocked out globally and not in region-specific manner. Whether these changes are the result of loss of *Per2* in neurons of the SCN, other brain sites or via a peripheral feedback remains to be determined.

Disruption in HPA axis regulation can be observed in multiple ways. First, the expected circadian rise in CORT observed at the onset of the active period (ZT12 in our current study) typically seen in WT mice is not present in *Per2−/−* mice. It is unlikely that this lack of diurnal CORT rise is due to a total shutdown of the HPA axis since we found enhanced increase in CORT following restraint. Thus, our studies are consistent with the hypothesis that the circadian clock plays a critical role in directing and ensuring the diurnal rise of CORT. When disrupted by the knockout of the Per2 gene, the diurnal CORT rise is attenuated. Interestingly, although there is a disrupted circadian rise, there is a prolonged increase in serum CORT levels in response to a transient mild restraint. Indeed, others have reported a connection between the circadian system and the HPA axis where various studies have shown elevated glucocorticoid levels, a shortened circadian cycle and disrupted diurnal CORT [6, 12, 24, 25]. This may suggest that with the disruption of circadian CORT rise, the HPA axis is hyperreactive resulting in a heightened and prolonged response to mild restraint. We show that the elevation in restraint-induced CORT secretion of *Per2−/−* mice is coupled to increased adrenal *Mc2R* mRNA expression, but not pituitary *Crfr1* mRNA levels. Thus, these data reveal that alterations at the level of the adrenal cortex, but not the pituitary, may underlie the enhanced secretory response to restraint in *Per2−/−* mice. There are many possibilities as to why a potential disruption in circadian rhythm may enhance CORT secretion. First, the enhanced secretory response may be due to changes in secretion of ACTH or proopiomelanocortin posttranslational processes at the level of the pituitary. Alternatively, the prolonged response to restraint may be due to alterations in the negative feedback control of CORT or even changes in the sympathetic projections from the hypothalamus via the splanchnic nerve which innervates the adrenal glands. Additionally, further studies are needed to determine potential rhythmicity of genes relevant to HPA axis regulation (such as *Mc2R* and *HSD1)* given that the adrenal is regulated by the circadian system [26]. Thus, it would be interesting to investigate adrenal and pituitary gene expression changes at multiple times in *Per2−/−* mice to more clearly understand underlying correlates of the observed alterations in glucocorticoid secretion. It would also be important to determine the potential role of sympathetic input to the adrenals.

In addition to altered CORT secretion, we also show that disruption to the circadian clock via *Per2* knockout resulted in deficits in startle response and increased depressive-like behaviors. In the ASR assay, *Per2−/−* mice had decreased startle response to a tone compared to WT mice. Additionally, in the PPI test, startle response in *Per2−/−* mice was disrupted when the difference between the pre-pulse and the startle tone was the greatest (i.e. 64/120dB group). Previous work has suggested a link between CORT and startle response [27]. For example, startle responses in rats peak during the active period and decrease throughout the inactive period when rodents have lower levels of endogenous CORT [28]. Therefore, the lack of diurnal CORT rise we observed in our study may explain why we observed a diminished startle response in *Per2−/−* mice during the inactive period. In addition to sensorimotor gating measurements, ASR/PPI behaviors are also linked to depressive-like phenotypes in that depressed patients show a decreased startle activity [29, 30]. The 5-HT_1β_ knockout mice which exhibit depressive-like phenotype has altered diurnal CORT rhythm [31], similar to what we found in *Per2−/−* mice. Therefore, the decreased startle responses we observed may be correlated to depressive-like behaviors. A further examination of startle responses during the active period is needed.

We also evaluated animals for depressive-like behaviors using the well-established FST [19]. In the FST, *Per2−/−* mice spend more time immobile (floating) and less time mobile (swimming/struggling) compared to WT controls. Previous work links dysregulation of both the circadian cycle and stress axis with depressive behaviors. Disrupted rhythms of the *Per2* gene, as well as other circadian genes, are correlated with major depressive disorder [32]. Our findings are consistent with these studies. Our finding of an enhanced HPA axis reactivity and increased depressive-like behavior is consistent with other studies [33–35]. Our study combines and expands on previous work to suggest that the vulnerability of developing depressive-like behaviors is related with dysregulation of the HPA axis. Further studies are needed to determine the underlying neurocircuitry involved in these behavioral changes.

Intriguingly, we did not observe increased anxiety-like behaviors in *Per2−/−* mice. In our study, *Per2−/−* mice spent more time in the center and less time in the periphery of the OFA compared to WT mice. In contrast, others have reported increased anxiety-like behaviors in *Per2* knockdown mice [36]. This discrepancy in findings may be a result of several factors. First, we utilized the OFA to determine anxiety-like behaviors while others have used elevated plus maze and light/dark box assays [36], potentially revealing anxiety-like traits that the OFA does not detect. Additionally, all of our behavioral assays were conducted during the second part of the light period (ZT730-ZT10). During this period, we also observed no differences in diurnal CORT secretion between WT and *Per2−/−* mice. This may account for why we unexpectedly observed less anxiety-like behaviors in *Per2−/−* mice. Others reported an abbreviated circadian period and altered locomotor rhythmicity in *Per2−/−* mice [9, 37]. Therefore, it is likely that behavioral testing during the dark period may render different behavioral results. Preliminary data in our lab (n = 3 WT, n = 10 *Per2−/−*) suggest that this may the case (data not shown). When tested in the OFA during the dark period (ZT16-ZT19), *Per2−/−* mice spent less time in the center (WT = 206.17 s versus *Per2−/−* = 591 s) and more time in the periphery (WT = 1425.1 sec versus *Per2−/−* = 1102.37 sec). Future studies are needed to fully assess anxiety-like behaviors during this alternate activity period and also expand measurements of CORT secretion, gene expression and depressive-like behaviors.

## Conclusions

Our studies showed that *Per2*−/− mice have a dysregulated HPA axis. The *Per2−/−* animals lack a diurnal CORT rise at the light-dark (inactive to active period) transition and have a prolonged, enhanced stressor-induced CORT levels. The *Per2−/−* mice exhibit depressive-like behaviors and deficits in startle responses and sensorimotor gating, all behaviors that are related to HPA axis dysregulation. That the *Per2* gene is conserved through phylogeny suggests its importance in regulating stress and stress-related behaviors in human and other species. Future studies are needed to determine how the circadian system may regulate the rhythmicity of the HPA axis.

## Data Availability

The datasets generated during the current study are available from the corresponding author (twu@usuhs.edu).

## Abbreviations

ACTH: Adrenocorticotropic hormone
ANOVA: Analysis of variance
ASR: Acoustic startle response
AVP: Arginine vasopressin
CORT: Corticosterone
CRF: Corticotropin-releasing factor
CRFR1: CRF receptor type 1
ELISA: Enzyme-linked immunosorbent assay
FST: Forced Swim Test
HPA: Hypothalamus-pituitary-adrenal
*Mc2R*: Melanocortin receptor 2
OFA: Open field activity
*Per2*: Period Circadian Regulator 2 (*Per2*)
PPI: Pre-pulse inhibition
PTSD: Post-traumatic stress disorder
PVN: Paraventricular nucleus
RNA: Ribonucleic acid
SCN: Suprachiasmatic nucleus of the hypothalamus
WT: Wild-type

## References

[1] Jacobson L (2014). Hypothalamic-pituitary-adrenocortical axis: neuropsychiatric aspects. Compr Physiol.

[2] de Kloet CS, Vermetten E, Geuze E, Kavelaars A, Heijnen CJ, Westenberg HG (2006). Assessment of HPA-axis function in posttraumatic stress disorder: pharmacological and non-pharmacological challenge tests, a review. J Psychiatr Res.

[3] Oyola MG, Handa RJ (2017). Hypothalamic-pituitary-adrenal and hypothalamic-pituitary-gonadal axes: sex differences in regulation of stress responsivity. Stress.

[4] Dallman MF, Jones MT (1973). Corticosteroid feedback control of ACTH secretion: effect of stress-induced corticosterone ssecretion on subsequent stress responses in the rat. Endocrinology.

[5] Handa RJ, Weiser MJ (2014). Gonadal steroid hormones and the hypothalamo-pituitary-adrenal axis. Front Neuroendocrinol.

[6] Nader N, Chrousos GP, Kino T (2010). Interactions of the circadian CLOCK system and the HPA axis. Trends Endocrinol Metab.

[7] Dickmeis T (2009). Glucocorticoids and the circadian clock. J Endocrinol.

[8] Oster H, Challet E, Ott V, Arvat E, de Kloet ER, Dijk DJ, Lightman S, Vgontzas A, Van Cauter E (2017). The functional and clinical significance of the 24-hour rhythm of circulating glucocorticoids. Endocr Rev.

[9] Zheng B, Larkin DW, Albrecht U, Sun ZS, Sage M, Eichele G, Lee CC, Bradley A (1999). The mPer2 gene encodes a functional component of the mammalian circadian clock. Nature.

[10] Bae K, Jin X, Maywood ES, Hastings MH, Reppert SM, Weaver DR (2001). Differential functions of mPer1, mPer2, and mPer3 in the SCN circadian clock. Neuron.

[11] Reddy TE, Pauli F, Sprouse RO, Neff NF, Newberry KM, Garabedian MJ, Myers RM (2009). Genomic determination of the glucocorticoid response reveals unexpected mechanisms of gene regulation. Genome Res.

[12] Yang S, Liu A, Weidenhammer A, Cooksey RC, McClain D, Kim MK, Aguilera G, Abel ED, Chung JH (2009). The role of mPer2 clock gene in glucocorticoid and feeding rhythms. Endocrinology.

[13] Russell AL, Richardson MR, Bauman BM, Hernandez IM, Saperstein S, Handa RJ, Wu TJ (2018). Differential responses of the HPA axis to mild blast traumatic brain injury in male and female mice. Endocrinology.

[14] Russell AL, Handa RJ, Wu TJ (2018). Sex-dependent effects of mild blast-induced traumatic brain injury on corticotropin-releasing factor receptor gene expression: potential link to anxiety-like behaviors. Neuroscience.

[15] Bauman BM, Buban KN, Russell AL, Handa RJ, Wu TJ (2019). Isoflavones alter hypothalamic-pituitary-adrenal axis response following photoperiod alteration. Neuroscience.

[16] Livak KJ, Schmittgen TD (2001). Analysis of relative gene expression data using real-time quantitative PCR and the 2(-Delta Delta C(T)) Method. Methods.

[17] Curzon P, Zhang M, Radek RJ, Fox GB. The behavioral assessment of sensorimotor processes in the mouse: acoustic startle, sensory gating, locomotor activity, rotarod, and beam walking. In: Buccafusco JJ, editor. Methods of behavior analysis in neuroscience*. *Boca Raton (FL); 2009. 21204341

[18] Faraday MM, Grunberg NE (2000). The importance of acclimation in acoustic startle amplitude and pre-pulse inhibition testing of male and female rats. Pharmacol Biochem Behav.

[19] Can A, Dao DT, Arad M, Terrillion CE, Piantadosi SC, Gould TD (2012). The mouse forced swim test. J Vis Exp.

[20] Lavebratt C, Sjoholm LK, Partonen T, Schalling M, Forsell Y (2010). PER2 variantion is associated with depression vulnerability. Am J Med Genet B Neuropsychiatr Genet.

[21] Rao R, Androulakis IP (2019). The physiological significance of the circadian dynamics of the HPA axis: interplay between circadian rhythms, allostasis and stress resilience. Horm Behav.

[22] Cascio CS, Shinsako J, Dallman MF (1987). The suprachiasmatic nuclei stimulate evening ACTH secretion in the rat. Brain Res.

[23] Abe K, Kroning J, Greer MA, Critchlow V (1979). Effects of destruction of the suprachiasmatic nuclei on the circadian rhythms in plasma corticosterone, body temperature, feeding and plasma thyrotropin. Neuroendocrinology.

[24] Dallmann R, Touma C, Palme R, Albrecht U, Steinlechner S (2006). Impaired daily glucocorticoid rhythm in Per1 (Brd) mice. J Comp Physiol A Neuroethol Sens Neural Behav Physiol.

[25] Pilorz V, Steinlechner S, Oster H (2009). Age and oestrus cycle-related changes in glucocorticoid excretion and wheel-running activity in female mice carrying mutations in the circadian clock genes Per1 and Per2. Physiol Behav.

[26] Stroth N, Kuri BA, Mustafa T, Chan SA, Smith CB, Eiden LE (2013). PACAP controls adrenomedullary catecholamine secretion and expression of catecholamine biosynthetic enzymes at high splanchnic nerve firing rates characteristic of stress transduction in male mice. Endocrinology.

[27] Miller MW, Gronfier C (2006). Diurnal variation of the startle reflex in relation to HPA-axis activity in humans. Psychophysiology.

[28] Chabot CC, Taylor DH (1992). Circadian modulation of the rat acoustic startle response. Behav Neurosci.

[29] Allen NB, Trinder J, Brennan C (1999). Affective startle modulation in clinical depression: preliminary findings. Biol Psychiatry.

[30] Kaviani H, Gray JA, Checkley SA, Raven PW, Wilson GD, Kumari V (2004). Affective modulation of the startle response in depression: influence of the severity of depression, anhedonia, and anxiety. J Affect Disord.

[31] Sollars PJ, Weiser MJ, Kudwa AE, Bramley JR, Ogilvie MD, Spencer RL, Handa RJ, Pickard GE (2014). Altered entrainment to the day/night cycle attenuates the daily rise in circulating corticosterone in the mouse. PLoS One.

[32] Li JZ, Bunney BG, Meng F, Hagenauer MH, Walsh DM, Vawter MP, Evans SJ, Choudary PV, Cartagena P, Barchas JD (2013). Circadian patterns of gene expression in the human brain and disruption in major depressive disorder. Proc Natl Acad Sci USA.

[33] Chen F, Zhou L, Bai Y, Zhou R, Chen L (2015). Hypothalamic-pituitary-adrenal axis hyperactivity accounts for anxiety- and depression-like behaviors in rats perinatally exposed to bisphenol A. J Biomed Res.

[34] Holsboer F, Ising M (2010). Stress hormone regulation: biological role and translation into therapy. Annu Rev Psychol.

[35] Ordyan NE, Pivina SG, Rakitskaya VV, Akulova VK (2016). Activity of hypothalamic-pituitary-adrenal axis of prenatally stressed male rats in experimental model of depression. Zh Evol Biokhim Fiziol.

[36] Spencer S, Falcon E, Kumar J, Krishnan V, Mukherjee S, Birnbaum SG, McClung CA (2013). Circadian genes Period 1 and Period 2 in the nucleus accumbens regulate anxiety-related behavior. Eur J Neurosci.

[37] Miki T, Chen-Goodspeed M, Zhao Z, Lee CC (2013). Circadian behavior of mice deficient in PER1/PML or PER2/PML. J Circadian Rhythms.

